# Artificial Intelligence for Food Safety and Quality Assurance: Technologies, Applications and Future Directions

**DOI:** 10.1002/fsn3.72377

**Published:** 2026-09-17

**Authors:** Harsh B. Jadhav, Moumita Das, Arpita Das, Aayeena Altaf, Kamal Alaskar, Robert Mugabi

**Affiliations:** ^1^ Food Technology, Amity Institute of Biotechnology Amity University Jaipur Rajasthan India; ^2^ Department of Food and Nutrition Swami Vivekananda University Barrackpore West Bengal India; ^3^ Department of Food and Nutrition Brainware University Kolkata West Bengal India; ^4^ Department of Computer Applications Bharati Vidyapeeth, Institute of Management Kolhapur India; ^5^ Department of Food Technology and Nutrition Makerere University Kampala Uganda

**Keywords:** artificial intelligence, blockchain, food safety, machine learning, quality assurance

## Abstract

Food quality and safety are critical components of consumer welfare. Food processing firms have been compelled to implement innovative measures to ensure food safety due to growing consumer demand for safe food. One new technology that has been applied in the food processing industry is artificial intelligence. Artificial intelligence‐based technologies, such as machine learning, blockchain, robotics, computer vision, and sensor networks, assist in identifying pathogens in food, detecting early outbreaks, and cleaning food processing equipment during processing and storage. Furthermore, the use of these technologies to enhance food safety has been driven by the goal of achieving a sustainable food system. The primary focus of this review is the application of AI‐based technology to enhance food safety and quality assurance.

## Graphical abstract

AI can help improve food safety and quality management by providing real‐time monitoring throughout the farm‐to‐fork supply chain, so that food producers and regulatory authorities are proactive rather than reactive in response to food contamination, and machine learning, computer vision, Internet of Things (IoT) sensors, robotics, natural language processing, and blockchain are technologies that can support real‐time detection of food contamination, quality inspection, outbreak prediction, equipment hygiene monitoring, regulatory compliance, and product traceability. These technologies can also improve decision making and mitigate the risk of human error and food waste, ensuring the delivery of safe and high‐quality food; however, the wider adoption of AI in food safety and quality management will require reliable data, validated systems, protection from cyber attacks, transparent governance, and appropriate regulatory frameworks.

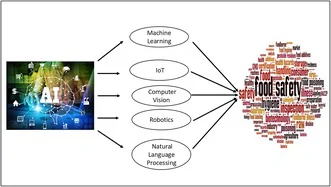

## 1 Introduction

The food processing sector is continuously working on developing strategies to ensure the delivery of safe food to consumers. During food processing, hygiene and quality control are crucial components of food and nutritional security. Over 10% of people are susceptible to food‐borne illnesses, and eating contaminated food is thought to be the cause of over 4.2 million deaths worldwide, according to estimates from the World Health Organization (WHO) (Li et al. 2023). As a result, customers are demanding safe and nourishing food and are becoming more concerned about food safety. Laboratory analysis, which is typically time‐consuming and prone to human error, is a component of the traditional procedures used to verify the safety and quality of food (Palakurti 2022). AI has the potential to overcome the limitations of traditional food quality methodologies. With the emergence of the Industrial 4.0 revolution, artificial intelligence has been applied in the food processing sector to ensure high productivity, nutritious food, food safety, and waste management (Ikram et al. 2024).

Artificial intelligence can assist in ensuring higher food safety and delivery of quality food to the consumer by application of Internet of Things devices, machine learning algorithms, and computer vision systems that can identify, assess, and control food quality and safety‐related concerns (Karanth et al. 2023). However, there is a need to incorporate artificial intelligence‐based techniques in the food processing and agriculture sectors to prioritize food safety issues. This will not only help in addressing the food safety issue but will also help in maintaining the high food standards laid down by legislative bodies across the globe. Artificial intelligence (AI) is visualized as the capability of robots or computer‐based programs to perform human tasks (Qian et al. 2023). In a recent study by Murphy et al. (2021), artificial intelligence‐based technology was used to identify quality practices that affect milk quality after pasteurization. The authors used bacterial spoilage indicator data to determine the parameters related to post‐pasteurization milk contamination. The use of sensor‐based devices is also an application of artificial intelligence in ensuring food safety. Devices such as the e‐nose and e‐tongue imaging are well reported in the literature for their use in safety assessment and ensuring food safety (Górska‐Horczyczak et al. 2016; Loutfi et al. 2015; Othman et al. 2023; Tan and Xu 2020). Literature also reports the use of AI‐based technology to detect fraudulent ingredients in food products, which is a major food safety concern (Haiminen et al. 2019). AI‐based devices are also used to predict the time/location that will have higher chances of pathogen growth or when there is a greater risk of contamination with pathogens, posing a risk to food safety (Qian et al. 2023). Furthermore, to strengthen the food safety system, AI‐based technologies can also be employed to ensure the proper cleaning of food processing instruments. Sensor technologies, based on the use of electrical, ultrasonic, and optical sensors, are designed to monitor the proper cleaning of processing equipment in the food industry (Escrig et al. 2019, 2020; Simeone et al. 2020; Yang et al. 2018). Owing to the numerous advantages of AI‐based techniques, the food industry has started adopting these technologies to meet the increasing demand for safe and nutritious foods. To assist this, the present review focuses on the fundamental application of AI‐based technologies to ensure higher food safety and quality assurance.

## 2 Importance of Food Safety and Quality Assurance

The primary way that Food safety safeguards the general public's health is by preventing foodborne illnesses. According to estimates from the World Health Organization (WHO), foodborne infections affect people of all ages. However, they are particularly dangerous for vulnerable populations, such as children, the elderly, and those with weakened immune system (WHO 2020). *Salmonella*, *E. coli*, and *Listeria* are among the organisms that cause the majority of foodborne illnesses and can have serious health consequences, including chronic disease and death. Effective food safety practices, such as proper handling, cooking, and storage, are crucial for lowering the risk of contamination and preventing public health catastrophes (Havelaar et al. 2015). Ensuring food safety and quality is essential to preserving consumer trust. Customers have high expectations for product quality and food safety in a competitive market. Trust and brand loyalty increase when food products are safe to eat and continuously fulfill the quality criteria. However, failures in food safety or quality can result in serious consequences, such as product recalls, harm to food firms' reputations, and financial losses (Sperber 2016). Customers are reassured that food items are safe and dependable by a well‐established quality assurance system that guarantees consistency and transparency throughout the food production process, boosting brand reputation. Food safety and quality assurance have major economic impacts on the food industry. Effective safety procedures reduce the likelihood of product recalls, which can lead to costly legal fees, limited market access, and reputational damage. Violating food safety regulations may result in penalties and criminal prosecution, which may have a serious financial impact (Tariq et al. 2020). As the global food trade grows, adhering to internationally recognized standards for food safety and quality is essential for increasing market reach, maintaining competitiveness, and enhancing economic sustainability. Those who operate in the food industry are required to abide by regulations controlling food safety and quality. Regulatory organizations such as the U.S. Food and Drug Administration (FDA), Codex Alimentarius Commission, and European Food Safety Authority (EFSA) guarantee the quality and safety of food. These organizations provide stringent guidelines and criteria that food companies must follow to prevent contamination and maintain product integrity (WHO 2020). Fines, product recalls, and restrictions on sales in particular regions are examples of serious consequences for non‐compliance. The importance of regulatory compliance is highlighted by the fact that the global food trade depends on uniform safety and quality standards. Food exporting nations must adhere to the regulatory requirements of their import markets (Tariq et al. 2020).

Recently, the significance of sustainability and ethical sourcing as components of food safety and quality management has increased. Customers are increasingly concerned about a product's environmental impact and ethical production practices, in addition to food safety. Quality assurance systems are being integrated with sustainability activities, such as reducing food waste, ensuring traceability, and promoting the humane treatment of animals and workers (Sperber 2016). Companies that prioritize sustainability, ethical conduct, and transparency in their food production processes enhance their reputation and build long‐lasting relationships with consumers. The trend towards sustainable food production is becoming an increasingly important part of food safety and quality, reflecting larger cultural movements towards environmental responsibility and ethical consumption.

## 3 Challenges in Ensuring Food Safety and Quality

Despite significant progress in food safety and quality assurance, the food industry faces a number of challenges in ensuring that food products are consistently nutritious, safe, and of high quality. These challenges are caused by various factors, including the globalization of food supply chains, shifting consumer preferences, technological advancements, complex laws, and environmental concerns. To address these issues, ongoing innovation, collaboration, investment in food safety protocols, and quality control techniques are required. The growing intricacy and globalization of food supply chains are among the main obstacles to guaranteeing food safety and quality. Food goods are more likely to become contaminated and lose quality at different points in the supply chain when transported across several nations and continents. Contaminants can enter the food chain at any stage from agricultural production to processing, packaging, and distribution (Tariq et al. 2020). For instance, contamination at farms in one nation may cause foodborne illness outbreaks that impact consumers across several regions. Maintaining uniform safety and quality standards in such international supply networks has become extremely difficult, particularly given the disparities in laws and enforcement capacities among nations (Sperber 2016).

The food industry is impacted by shifting consumer expectations. The growing demand for convenient, ready‐to‐eat, and longer‐lasting foods requires food producers to innovate while preserving product safety and quality. Additionally, consumers are increasingly choosing meals that are responsibly produced, organic, and free of artificial additives (Sperber 2016). Because many of these products are more likely to become contaminated or expire without additives or preservatives, it can be challenging to satisfy these requirements while maintaining safety rules. To find a balance between these needs and food safety laws, firms must invest in novel technologies, such as natural preservatives or modified packaging. Although there is great promise for improving food safety and quality control through technological improvements, there are a number of obstacles to overcome before their implementation. Food safety and uniformity can be increased by utilizing technologies such as blockchain for traceability, artificial intelligence (AI) for tracking food safety risks, and automation in processing plants. However, these technologies frequently require infrastructure, specialized knowledge, and large financial investments (Tariq et al. 2020). Food firms might also have trouble incorporating these technologies into their current systems and ensuring that they are correctly used across the supply chain. Because data breaches or cyberattacks could jeopardize food safety records and have dire repercussions, it is also crucial to ensure the security and integrity of the data produced by these technologies. Navigating intricate and frequently contradictory regulatory systems that control food safety and quality requirements in many jurisdictions presents another formidable obstacle. Despite the considerable uniformity provided by international organizations, such as the Codex Alimentarius Commission, there are still differences across countries' food safety laws, policies, and enforcement systems. For businesses that operate in several markets, disparities in regulatory frameworks can be confusing, making it challenging to meet all the different criteria (World Health Organization [WHO] 2020). Additionally, companies must constantly modify their procedures to comply with food safety rules as they react to new scientific findings and emerging dangers. A proactive strategy to monitor and apply new standards is necessary because of the constantly shifting regulatory landscape, which can put strain on resources, especially for smaller companies. Food safety and quality are seriously threatened by environmental factors and climate change. Food safety may be affected by shifting weather patterns that affect crop growth, pest prevalence, and livestock disease transmission (Havelaar et al. 2015). Floods and droughts are examples of extreme weather events that can interfere with food production and transportation, resulting in shortages, contamination risks, and deterioration of quality. Moreover, chemical pollutants may enter the food supply because of the use of fertilizers and pesticides in response to these environmental stressors. Food producers must adjust to these environmental changes while upholding safety regulations, which frequently call for modifications to production methods, farming practices, and the adoption of new safety procedures. Ensuring that employees are properly taught and equipped to conduct food safety procedures is a less talked‐about but no less significant difficulty in food safety and quality evaluation. Defects in food products and foodborne infections are still largely caused by human error. According to Sperber (2016), improper handling, storage, or processing can cause food products to become contaminated, deteriorate, or contain foreign elements. One of the ongoing challenges is to ensure that workers at every stage of the food production process receive comprehensive training on food safety regulations and are diligent in implementing them. To lower the possibility of human mistakes, businesses need to invest in ongoing training initiatives, quality assurance inspections, and a safety‐oriented culture.

Financial limitations prevent many food producers, particularly smaller companies, from investing in cutting‐edge food safety technologies or upholding strict quality assurance procedures. It may be unaffordable for small and medium‐sized businesses (SMEs) to develop cutting‐edge food safety systems, carry out routine testing, and adhere to changing regulatory requirements. Food safety and quality may be compromised because of small businesses' inability to compete with bigger rivals (Tariq et al. 2020). Additionally, the need to decrease expenses and boost profit margins can occasionally result in a lack of attention to safety and quality procedures, which, despite short‐term profitability, can have long‐term negative impacts on consumer health and product safety.

## 4 Role of AI in Enhancing Food Safety and Quality Assurance

Artificial Intelligence is an ingenious tool that imitates the intelligence and aptitude of humans using various technologies (Palakurti 2022). These tools can provide advice and insights to assist farmers in making decisions and actions. The general utilization of AI addresses food security and makes a significant contribution to the field of food technology. The use of AI has benefited from different potential methods for food‐based businesses, such as providing accurate calculations and meticulous decisions. AI technology uses algorithms to calculate the degree of performance that helps to determine the patterns and also detects the foreseen issues or events to reduce several problems and challenges that, in turn, enhance and improve the quality and security of food (Chhetri 2024). Sensors that are enabled by AI can evaluate the quality of soil and indicate plant diseases, which helps manage timely solutions. Advanced forms of AI can elevate the productivity of agricultural products and provide better distribution with proper and skilled planning. Moreover, AI enhances safety and security by continuously monitoring the levels of contamination and adhering to food safety rules and regulations of food safety. These types of technologies will enhance the reduction of food waste and ensure a sustainable global supply. The efficient distribution of fresh produce by diversifying the delivery destinations can be ensured by AI, which can enhance shelf life and preserve quality. The main role of AI is to change the predictions of food quality in terms of food science, technology, and agriculture. The main hurdles present in observing food quality and expiry numbers are time‐consuming investigations and sample destruction. The digital revolution has revealed that AI exists as a compelling analytical means for determining the quality of different agricultural and food products (Ali Eltabey 2023). An artificial neural network (ANN) is the most commonly adopted AI technology that can be used in assessing the quality of foods. This system is a mathematical model based on nodes of both non‐linear and linear processing characteristics (Kudashkina et al. 2022). The main advantage of an ANN is its ability to predict the foundation of collateral reasoning. Several studies have shown that there are many potential advantages of utilizing ANN to measure the quality of foods and other agricultural products, such as coconuts, potatoes, and olive oil. Several researchers predicted the dried content of fruits, such as mangoes, by using ANN and obtained superior results in all aspects. Enhancing global food safety standards through international collaboration and policy harmonization. According to Abdel Qader (2023), the nutritional values of fried fish, such as time, temperature, and oil content, were restored using the AI intelligence (ANN) technique. Customer awareness and health concerns are growing daily. Therefore, the evolution of AI provides a platform for easy tracking of the quality of different foods and agri‐based products. Advanced forms of AI will enhance and optimize the output and proper distribution to predict the demand for food supply, the process of distribution, and other dynamics of the food supply chain, and determine the preferences of the population (Vegesna et al. 2024). Addressing different challenges in AI‐enabled food quality management is the most crucial factor for enhancing the quality and safety of foods. The quality of food systems, such as cafes, hotels, outlets, and online food delivery systems, is improved and enhanced by AI combined with data science via several algorithms that can help in better sales forecasts. AI also helps manage the risk of agricultural supply, particularly in situations such as the COVID‐19 Pandemic. The combination of different AI techniques, such as expert systems with cloud computing, with the intelligence of business, data mining, and machine learning, has significantly enhanced the quality of all aspects of food quality management by decreasing errors and inaccuracy in accounting information (Nayak and Dutta 2023). The utilization of AI has enhanced and improved the experiences of customers by managing the supply chain more effectively, and has also helped to find new, low‐budget methods to range and serve customers. With improved technologies, AI has reduced the risk of production and quality in the field of food processing. Different innovations that can sustain the maximum usage of resources will help eliminate the errors made by humans, which will further lead to maximum savings and developed operational processes. AI techniques have been proven to maintain and enhance quality control, leading to higher yields of the final products, thereby decreasing the impact on the environment (Nychas et al. 2016). Operational costs in the food processing industry can be greatly reduced by using a combination of artificial intelligence. AI can also help in differentiating food supplies, observing different standards of hygiene, and thus reduce and prevent contamination, which in turn helps reduce expenses (Ajiga et al. 2024). These characteristics of AI helped reduce the high cost of processing techniques of operation by analyzing the consumption of energy as well as investment in hardware materials. Owing to several advantages, AI will be utilized more by food business owners because it has many benefits regarding the utilization of money, energy, etc. Improved quality in the field of processing, manufacturing, and traceability is achieved using AI, with better results in traceability, logistics decision‐making, precision, and accuracy. Various difficulties in the agricultural sector have been rectified, and this has improved the supply, leading to the best innovative secured food operations (Karanth et al. 2023). After harvesting fruits and vegetables, AI has been introduced to help retain and maintain freshness, which helps reduce post‐harvest losses. Cognitive approaches and the optimization of AI markedly improve the management of processing and production. Different systematic weaknesses still exist in food management systems that can be addressed by using AI to achieve better competitive, sustainable, and productive outcomes. The utilization of AI in taste profiling provides many advantages because it improves the overall qualities, including flavor and taste, of food materials such as aged dry beef, as shown in Table 1; it helps in the identification of compounds that contain critical flavor. Therefore, AI can enhance the sensory properties that help in the classification of taste profile compounds. Several AI scientists and researchers can help in the assessment of flavors in foodstuffs such as tomato sauce and garlic flavors. Therefore, information on consumer acceptance and preferences can be derived (Zhou et al. 2022). Another main parameter that affects food acceptance in terms of all factors is texture. Food textures and quality with safety prediction models have been used in combination with ANN, which helps to develop better food with improved texture and quality, which will improve the safety of food. AI has also been introduced in food traceability systems, which improves quality assurance. AI and the Internet of Things (IoT) can be used for better food traceability and meticulously solve issues related to old traceability systems, such as incompetent reliability and complex data storage. It further creates seamless tracking between food items and provides different techniques for clarity and dependability of data regarding food safety and quality assurance. Experts have observed that food safety and quality assurance may be improved by incorporating AI techniques, which can provide the best and most accurate evaluations (Liu et al. 2023). Food safety and assurance are very important steps in traceability that allow different and advanced packaging techniques to help in analyzing and tracing quality data, such as freshness. Transparency and traceability are known as important parts of food safety and its regulations, by which information about the imbalance, restoring public policies with trust that will help in elevating the efficacy of the measures taken in food safety with confidence. Food safety and quality assurance play an important role in the sustainability of the agricultural‐based food supply market, highlighting the economic and market‐driven reasons driving traceability and its possible consequences on sustainability (Erkinjon and Feruz 2024). Food safety has been connected to the ability of a food traceability system to handle crises. It can also help in the determination and isolation of problems, as well as reduce the effect of food recalls. AI can potentially provide marked changes in different aspects of agricultural areas, such as maps of crop yields, forecasts of yields, and the management of personnel working in the field, which will help in the determination of several decisions and assistance to both farmers and producers. These developments provide better solutions in the food safety and assurance sector, leading to better sustainability in the market (Qian et al. 2023). The application of AI can improve the quality and safety of food, which encompasses factors such as accessibility, availability, and utilization. It can also be a very helpful tool for tracing food security on different social media platforms. Moreover, better advancements in the food business with ensured food security can be seen after using AI, and determining the data at a large scale can bring about sustainable advancements in quality assurance (Chamara et al. 2020).

**TABLE 1:** Role of Artificial Intelligence in the food safety of different foods.

Product	Objective	Type of industry	Essential output	References
Barley	Classification of its grains	Quality Control	The accuracy was classified by utilization of AI and was found to be above 73%, which was considered satisfactory	Kumar et al. (2021)
Coffee and Coffee beans	Determination of suitable process during utilization of dry mill and Determination of grading on the basis of quality	Production unit and sensory evaluation	AI was able to choose the process for a particular product. The accuracy value was 92%. The developed AI was able to find the best grade of coffee beans with an accuracy of 96%	Agnusdei et al. (2024)
Maize	Determination of diseases and concern pest	Agriculture	Detection of diseases and pests was found with an accuracy of 75% as well as AI provided ways to stop these diseases	Chamara et al. (2020)
Additives	Determination of rating of halal safety of additives	Food Safety	Consumers were able to find the nutritional value and halal safety rating	Fu (2021)
Animal products such as flesh and dairy products	To determine the performance of livestock depending on various factors	Production	Identification of best practices that are used to enhance the production of meat and milk products	Addanki et al. (2022)
Food products	To observe and predict the quality of the product during the process of production	Unit of quality control	The use of AI provides indicators that help to determine and monitor the quality of food products and enhance the quality of products as well in some cases, defects and problems were also detected	Sharma et al. (2021)
Fresh produce	Optimization of food quality	Food safety and sustainability	AI was able to develop better distribution methods that decreased the formation of carbon dioxide. It also provided the waste management system	Kakani et al. (2020)
Rice and soybean	To assist the farmers in decision‐making regarding their crops	Agriculture and production	Farmers were assisted in the process of selection of different seeds with the best quality, and different methods were suggested in tackling the diseases both in the case of rice and soybean	Chamara et al. (2020)

## 5 Machine Learning Techniques for Food Safety

Machine Learning (ML) is capable of providing a combination of different data, such as structured and unstructured data, which helps in the detection of food safety. The purpose of ML is to monitor and predict food safety factors (Deng et al. 2021). Currently, applications of ML are mainly focused on food safety related to pathogens that cause foodborne illnesses and help in tracing the origin of these illnesses. Different types of ML techniques use both structured and unstructured data that can help in the prediction of food safety, such as convolutional neural networks (CNN), which are used to determine image data. Different conventional methods were used on large scales, such as chromatography and spectroscopy; these provided perfect outcomes with better reproducibility but also had some limitations of long, time‐consuming processes, difficult sample preparations, and technical operations. On the other hand, ML techniques help in the recognition of biomolecular structures and conversion of downstream signals that provide the possibility of monitoring and assessing food safety. These techniques are much better than previous analytical methods. ML provides fast and efficient food safety, with better monitoring and assessment (Wang et al. 2022). It can also facilitate masking of suspicious samples using real‐time detection. Currently, ML helps detect the residues present in food products that originate from different agricultural practices, additives, pathogens, toxins, and other food contaminants.

ML can be categorized in different ways, based on several criteria. Based on the learning systems, ML can be divided into four categories: (a) supervised learning, (b) unsupervised learning, (c) semi‐supervised learning, and reinforcement learning. The information of samples provided in the form of labels is part of unsupervised learning; it also exposes the attributes and rules that are inherent in the data through samples without labels (Van den Bulk et al. 2022). Unsupervised learning has been utilized in the analysis of several social networks for the organization of computing clusters. Supervised ML is a technique used in the natural science sector. In this type, data and their input and output values are known, which helps create a specific relationship. This type of learning includes both classification and regression. Semi‐supervised ML comprises two types of learning: transductive learning and inductive learning. The extraction of information from external sources, as compared to a dataset in the case of reinforcement learning; these types of information are used for decision‐making. The goal of ML is to acquire knowledge about the environmental state of the behavior. Approval for this type of learning is provided through feedback in the environment. There are four main tasks executed by ML: classification, clustering, regression, and dimension reduction. The predictive problem in which a dataset is assigned to discrete classes is known as classification. In contrast, the problem arising from a continuous target value is known as regression. The spatial distribution and visual set of data belonging to one category is known as clustering. The dimensional reduction of variables to lower spaces is called the reduction of dimensions. Of these four types, regression and classification are included as parts of supervised learning that are utilized to develop prediction models to create results based on the given problems (Jiao et al. 2020). However, there is one difference: the target present in regression learning is continuous, while it is discrete in classification learning. There are different types of learning algorithms for classification, such as support vector machine (SVM), stochastic gradient descent algorithm (SGD), Bayesian estimation, ensemble, and K‐nearest neighbors. In addition, among these, a few can be used for regression, such as SVM and SGD. Analyzes of samples are performed by both clustering and classification, but the difference is that classification provides specific targets with particular labels. However, there were no labeled targets. In addition, classification is a supervised learning technique, whereas clustering is an unsupervised learning technique. In clustering, the attributes can be determined by the distribution of the samples. The most utilized algorithms for clustering include K‐means, Gaussian mixture model, and expectation–maximization (Zhou et al. 2019). The most important type of ML is dimensionality reduction, because it includes both types of learning (supervised and unsupervised). This involves changing redundancies to represent data in lower‐dimensional subspaces. Principal component analysis is the primary part of the dimension reduction algorithm, including some other algorithms such as partial least squares regression (PLSR) and linear discriminant analysis (LDA). The initial step of ML is the acquisition and formation of data to provide the input for the resulting learning that will serve as the causal factor for the model. This is a crucial step because it requires a large amount of data, followed by another step, which is the development of the model utilizing the training (Qian et al. 2023). Subsequently, a t‐appropriate algorithm is identified, which helps in the validation of the model. This validated model should be tested to make predictions using new data. Finally, the model was tuned to improve the performance of the algorithm. Additional data with many adjusted features and parameters are needed. Finally, the overall learning system must undergo an assessment of its accuracy. The efficiency and accuracy of food biosensors can be enhanced using suitable algorithms such as SVM, which can be used to find non‐linear and high‐dimensional data. Recently, biosensors have been developed for the detection of some antibiotics that are used in animal medicine, and SVM has been used to determine the concentration of these antibiotics. However, many other algorithms can be used to monitor food safety, such as the smartphone‐based lateral flow assay, which was used to differentiate the concentrations of *Salmonella spp* by determining the test line images using SVM and KNN (Niu 2024).

## 6 Computer Vision Application in Food Safety

Computer vision technology has been extensively utilized in the food safety sector, playing a crucial role in detecting and eliminating unwanted materials, such as animals, foreign objects, and soil from raw food products. With the rapid advancement of machine vision, nondestructive techniques for assessing various visual appearance aspects, such as color, texture, and shape, have become feasible and essential (Zhu et al. 2021). This progression has led to the development of sophisticated faultless inspection techniques that are instrumental in examining microbiological control and implementing safety standards for meat products, ready‐to‐eat meals, and canned and pouched packaged foods. As a result, image processing technology has emerged as a valuable tool, capable of assessing product quality to a significant extent and ensuring that food safety protocols are upheld. Numerous inspection systems have been developed for a variety of purposes, all of which aim to enhance. Food safety and the quality characteristics of products (Jin et al. 2020). Notable examples include vision‐based systems specifically designed for the classification of similar goods, thorough inspection of components, and effective recognition of organic products in a landscape increasingly focused on health and sustainability. It is important to recognize that food‐processing environments are typically classified as hazardous and can often present difficult and challenging conditions, where individuals might face considerable risks while working. Therefore, it is even more critical to rely on these advanced technologies to ensure safety and quality throughout the food supply chain (Guo et al. 2021).

Computer vision systems inherently provide invaluable capabilities for real‐time monitoring in various sectors. They facilitate continuous surveillance, offering 24/7 oversight, while simultaneously automating quality control processes that are essential in numerous industries. Specifically, the implementation of computer vision solutions in food processing and handling environments to execute assembly and packaging functions has led to numerous successful outcomes and remarkable case studies in practice (Kakani et al. 2020). Equipped with an array of multiple computer vision sensors, the automated robotic system proved to be highly effective, readily managing food and packaging products for precision and efficient sorting and packing. Another noteworthy application is the meticulous content inspection and removal of foreign materials from food products during processing. For instance, an intelligent machine vision system has been successfully implemented to produce roasted peanuts, focusing specifically on monitoring and isolating foreign materials, including harmful substances such as aflatoxin mold, which can affect food safety (Zhu et al. 2021). Through the deployment of selective spectral imaging techniques, advanced computer vision sensors can effectively distinguish between infected and normal grains, leading to a significant reduction in errors during the removal of valuable yet compromised materials. Furthermore, a sophisticated vision system integrated with various associated technologies can establish an automatic ejection system specifically designed for mushrooms, allowing the selection of multiple quality attributes (Ismail and Malik 2022). This is essential for maintaining high standards of food quality and safety. Beyond the production line, computer vision applications have been extended to monitor specific environments and processes. For example, a computer vision‐based system was proposed to observe and track the activity of honey bees in a controlled environment (Paul et al. 2022). This innovative system demonstrated the capability of performing semi‐automated measurements of several critical parameters, including coloration, temperature, and movement, thereby contributing valuable data to apidology research. In addition, computer vision solutions have been effectively applied along the packaging line to conduct thorough inspections of proper printing services for food wrapping. A particular system that performs additional precision checks, such as monitoring product seal integrity and headspace, was described and demonstrated using a cooperative industrial robot in conjunction with a high‐resolution sensor system. This collaboration shows that through the thoughtful integration of robotics and associated sensor technologies, the inspection processes of packed meat can be significantly enhanced and further expanded. It is increasingly apparent that the integrated utilization of computer vision alongside robotics, sensors, and other relevant technologies has gained recognition within the food and beverage industry as a strategic response to the ongoing challenges of the 21st century. These challenges include labor shortages, rising production costs, and stringent regulatory demands (Zhu et al. 2021). Moreover, it has been convincingly demonstrated that advanced computer vision systems are capable of generating substantial competitive advantages for stakeholders within the industry. By offering enhanced productivity, improved process control, greater customer satisfaction, and additional opportunities for the innovative development of new food products, computer vision technology is a transformative force in the field (Khan and Al‐Habsi 2020).

## 7 Natural Language Processing in Quality Assurance

**FIGURE 1:**
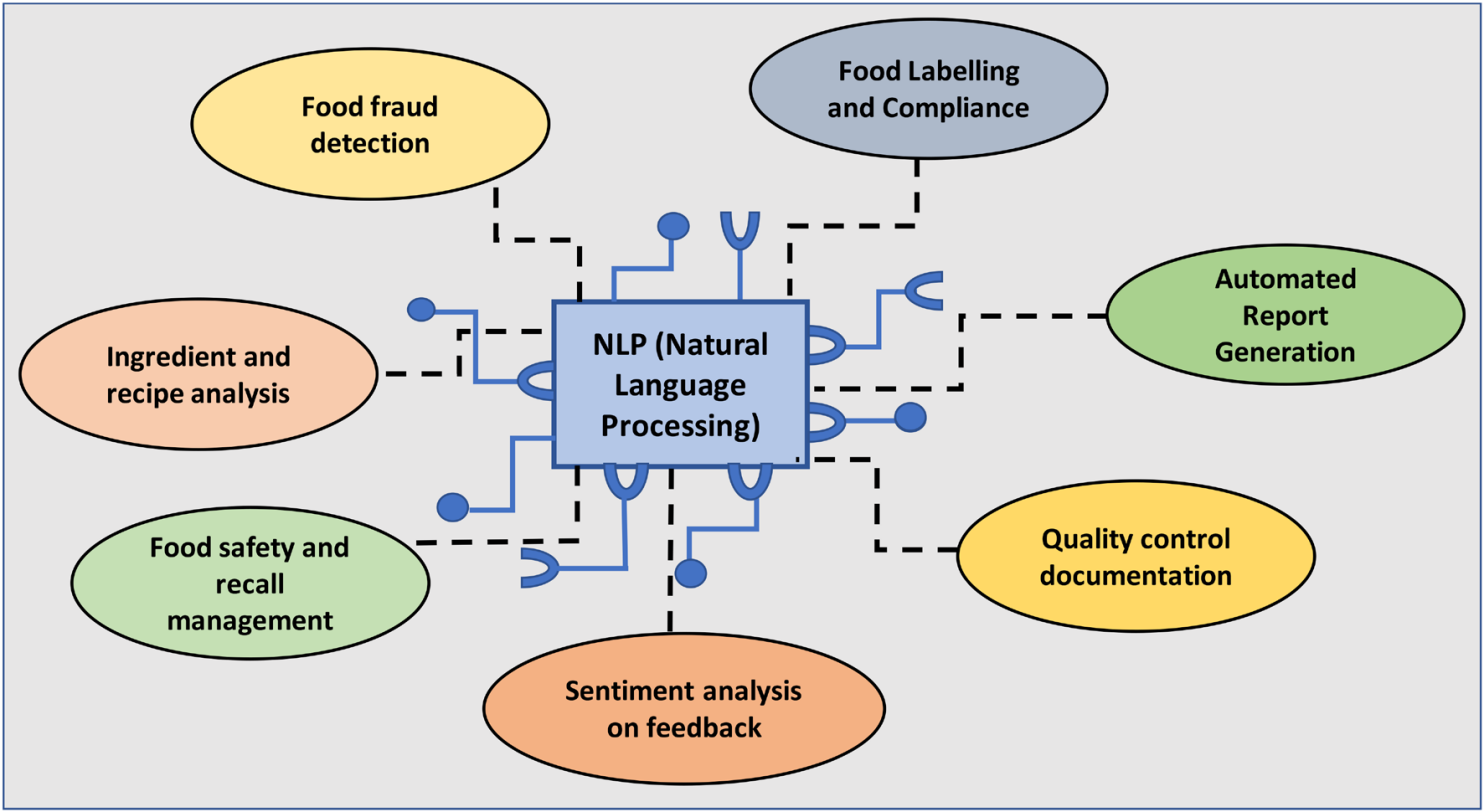
Natural Language Processing (NLP) contributes to quality assurance in food.

The food industry faces many challenges in ensuring the safety and quality of its products within the supply chain. To improve a company's strategic position and operational efficiency, quality management systems traditionally focus on satisfying customer expectations and quality targets. However, the complexity of food production and distribution has increased, necessitating the development of advanced quality assurance methods. One topic that has generated much interest is the use of natural language processing techniques to guarantee food quality. Natural language processing is an area of artificial intelligence that examines interactions between computers and human language. Over the past 10 years, natural language processing has made significant strides, evolving from a specialist field to a crucial tool in practically every industry that consumes linguistic data, including the food industry. Natural language processing, which is crucial for comprehending context‐dependent data generated throughout the food quality assurance process (Figure 1), has enabled machines to perceive, interpret, and analyze human language (Torfi and Associates 2020). The food industry generates vast amounts of unstructured data, such as regulatory reports, equipment maintenance logs, and customer feedback.

According to recent surveys, pre‐trained natural language processing (NLP) models considerably improve the accuracy and efficiency of text‐based categorization tasks. More complicated classifications are now achievable because of the integration of attention mechanisms into natural language processing (NLP), which has greatly improved the ability to extract contextual information from food product descriptions. These advancements are particularly relevant to food classification because product labels usually use complex and varied wording. NLP's ability to process vast amounts of textual data quickly makes it an appealing alternative to manual categorization methods. However, although these models have been successfully used in a variety of fields, their application in food classification has received little attention. Further research is needed to adapt the existing NLP algorithms to the intricacies of dietary data. Given the industry's reliance on vast amounts of documentation, such as regulatory standards, inspection reports, and customer feedback, natural language processing (NLP) provides a more effective way to manage and extract insights from several data sources. This section examines the use of NLP in food quality assurance, focusing on compliance monitoring, sentiment analysis, predictive analytics, traceability, and risk assessment. Ensuring regulatory compliance is essential for food quality assurance to uphold safety and fulfill legal obligations. NLP technologies make it easier to monitor compliance and reduce the possibility of human mistakes in document reviews by automating the extraction and validation of data from inspection reports, audit results, and government‐issued standards. To ensure that QA procedures follow set procedures, NLP systems can interpret complicated food safety laws, such as those issued by the Food and Drug Administration (FDA) or ISO (International Organization for Standardization) 22,000 norms. According to studies, NLP applications in regulatory compliance improve compliance tracking accuracy, freeing up resources for QA teams to concentrate on high‐priority non‐compliance regions (Bhatia et al. 2022). Analyzing consumer feedback is essential for understanding how consumers perceive the quality of food goods and identifying potential issues early on. Businesses can use sentiment analysis, an NLP technique, to process and assess customer attitudes in online reviews, social media posts, and survey responses. Sentiment analysis can help QA teams to understand recurring complaints, such as those concerning freshness, taste, or packaging, which could indicate a breach in quality standards. According to Sujatha and Mukherjee (2021), food companies can use sentiment analysis of customer feedback to respond proactively, increase customer happiness, and lower reputational concerns. Predictive quality analytics is made possible by fusing machine learning models with natural language processing (NLP), which helps companies anticipate issues in the product supply chain before they arise. By analyzing historical data on inspection records, quality assurance documents, and customer complaints, NLP‐driven predictive algorithms can identify patterns that may point to potential threats. These models reduce product recall and spoilage by assisting the QA teams in implementing preventive actions. Patel et al. (2020) claimed that NLP‐based predictive analytics have improved proactive quality management and reduced operating costs associated with product waste and quality failures.

Traceability and supplier verification are crucial components of quality assurance in the food industry, because multiple vendors may supply ingredients from different geographic locations. NLP techniques ensure that compliance documents, ingredient specifications, and certifications meet requirements by expediting supplier document processing. NLP systems may also trace products through the supply chain by creating an audit trail that links sources, certifications, and test results. In addition to facilitating speedier recall in the event of contamination, this function generally improves supply chain transparency. NLP has reduced the risks of sourcing and supplier inconsistency by improving traceability and compliance verification in supplier documentation management (Kumar et al. 2023). Early risk detection for food safety is greatly aided by NLP applications for real‐time monitoring. NLP systems enable businesses to respond swiftly to new hazards by continuously monitoring social media, news sources, and government alerts for mentions of foodborne diseases, contamination incidents, and recalls. Food firms may take prompt action, including recalling impacted batches or sending warnings, owing to this real‐time knowledge. According to Tran et al. (2021), real‐time risk detection powered by natural language processing (NLP) has improved consumer safety and confidence by reducing reaction times for food safety issues. These NLP applications not only expedite the quality assurance process but also provide a proactive approach to food safety, ensuring that products meet the highest standards for consumer satisfaction and health. As NLP tools advance and are being combined with other technologies, including machine learning and the Internet of Things, more advancements in quality assurance in the food industry are expected.

## 8 Sensor Technologies and IoT in the Food Industry

Sensors, in their most basic and primitive form, have been employed during diverse stages of food production since the 1950s. These sensors have evolved significantly over the years, becoming not only more precise but also increasingly affordable and rugged, making them highly valuable in real‐life food production processes (Yu et al. 2022). Today, a multitude of different types of electronic systems, commonly referred to as smart sensors, are utilized in food production and across various stages of the distribution process. Among these advanced systems are extraordinary devices such as the e‐nose and e‐tongue, which have been engineered specifically for food‐related applications (Pal and Kant 2020). These sophisticated sensors were designed to enable seamless and intelligent integration with computer‐based systems, thereby enhancing the overall safety and quality standards of food products. Unlike traditional systems that collect subjective and often unreliable data, modern smart sensors can accurately represent the actual conditions of a product (Zuo et al. 2022). They achieve this by meticulously collecting critical data, such as temperature, humidity, and aroma. Furthermore, smart sensors possess advanced capabilities that allow them to gather and analyze sensor outputs effectively, subsequently reporting sensory events as electronic signals to other integrated systems for enhanced monitoring and control (Chen et al. 2020; Zhang et al. 2022).

The tremendous potential of Internet of Things (IoT) devices to deliver real‐time data concerning various environmental factors, such as temperature, humidity, and other similar aspects that could significantly influence the condition and quality of food, is one of the critical elements in predicting possible food spoilage. This advanced technology enables storage operators to maintain optimal and appropriate storage and transit conditions, which is crucial (Jagtap et al. 2021). Additionally, leveraging the data gathered from IoT devices, manufacturers, and food quality assessors can monitor and examine the quality and shelf life of diverse batches of food products with much greater precision and frequency. Moreover, the integration of IoT technology offers the food industry a range of benefits not only in terms of products, but also in the realm of information and data management, both of which are continuously evolving and transforming to meet modern needs (Torres‐Sánchez et al. 2020). However, it is important to note that spoilage prevention is not the sole application of highly accurate temperature sensors. In situations where adverse quality effects have already manifested, a more sophisticated and real‐time understanding of these effects can be achieved through a detailed data analysis of the stored produce. This could help in identifying non‐visible or organoleptic characteristics of the produce that are correlated with, and could elucidate, any observed depreciation in quality resulting from unfavorable storage conditions (Javaid et al. 2021). Furthermore, this insightful data can assist storage managers in making well‐informed decisions regarding the necessary treatments and interventions to meet the desired and expected standards of produce quality. Given that IoT systems can be connected to the cloud, this connection provides an elevated and more precise level of informatization and resource sharing across the industry (Ben‐Daya et al. 2020). For instance, it greatly enhances food traceability, allowing for the improved tracking and monitoring of products throughout the supply chain. Data analytics will undeniably play a pivotal role in tapping into the potential of IoT. By employing IoT solutions alongside other Big Data‐enabled applications, the industry will be able to collect, organize, and analyze a wide range of information and monitor food safety both at the point of production and throughout the intricacies of supply chain management (Zhu et al. 2021). These innovative applications can significantly assist the food industry in making informed, data‐driven decisions. Notably, while challenges associated with consumer acceptance exist, primarily because individuals often favor possessing control over the information they receive from producers, interconnected sensors operating in the cloud could facilitate network systems in running serial numbers across different companies and batches of products (Lutz and Coradi 2022). This capability would be invaluable in establishing and pinpointing the source of spoilage and quality issues across the broader scope of the food chain, enhancing the overall efficiency and safety of food distribution (Nagarajan et al. 2022; Bigliardi et al. 2022).

## 9 Block Chain Technology in Food Traceability

Food Traceability is the function or ability to recall all data about the food product. According to the International Organization for Standardization (ISO) (ISO 22005:2007), food traceability is defined as the ability to follow the movement of a feed or food through specified stages of production, processing, and distribution (Sgroi 2022). The traceability system provides information about food origin and processes involved in both transportation and storage. An ideal food traceability system provides information on food products from farms to forks. The acquired information should be effective in terms of quantity and quality. Traceability is known to ensure food safety (Sri Vigna Hema and Manickavasagan 2024). During the traceability process, there are two types of data detection systems: one‐step‐forward, one‐step‐back, and an aggregated architecture. The step forward and step back process takes time to complete, while aggregated architecture data is incorporated, but it depends on the third parties. Thus, there is the possibility of another type of tracing system: a blockchain system. Blockchain is a type of system that allows integration of the system without interference from third parties; it also distributes data to each party involved in the supply chain. One type of blockchain, known as the Food Trail blockchain, is used to record and trace the transportation and transformation of food products in the supply chain (Khan 2022). The evaluation showed that the Food Trail Blockchain fulfilled the distributed, verified, and immutable aspects. The system has low performance in transaction handling related to breadth and depth aspects, owing to limited server capacity and complicated processes. However, the system has advantages related to access and precision because all transactions are verified, immutable, and stored locally at every node. A blockchain is a record that contains data and information about food products. A blockchain contains a data structure that is designed to assist the distribution of digital data, where information is stored safely in the form of chained blocks. A blockchain traceability system can establish a link of trust to track information among personnel involved in the process of food chain supply. It also includes real‐time monitoring, which is a vital aspect of food safety, particularly in logistics (Lin et al. 2019). The application of blockchain technology is very important, as it can help create a system that is decentralized, immutable, transparent, and reliable for real‐time decision‐making. Other tools are used with blockchain tractability systems, such as radio‐frequency identification. The utilization of blockchain systems is increasing daily and is considered an efficient and effective method of traceability systems in the field of food safety (Oriekhoe et al. 2024). A block‐chain system allows people to develop a digital register of transactions that can help share information among the participants in the supply chain. A blockchain can provide information to several parties through the supply chain. It can contribute significantly to the food ecosystem, such as the origin of products and ingredient tracking, thus improving the sustainability of food items with better food safety (Hao et al. 2020).

## 10 AI in Foodborne Illness Detection and Prevention

The identification and prevention of foodborne infections have been completely transformed by artificial intelligence (AI), which has made it possible to observe trends in massive data‐sets, anticipate outbreaks before they occur, and identify dangers early. AI allows public health organizations and food safety teams to take a proactive approach to managing foodborne illnesses because of its ability to process enormous volumes of data from many sources. Predictive analytics, outbreak identification, contamination tracing, environmental monitoring, and consumer health monitoring are some of the topics covered in this section's exploration of AI applications in foodborne illness detection and prevention. Using both historical and current data, predictive analytics, which are driven by machine learning models, enables the prediction of possible outbreaks of foodborne illnesses. Artificial intelligence (AI) systems can predict the times and places when the risk of foodborne illness may be higher by examining variables such as temperature fluctuations, seasonal trends, and geographic risk patterns. Public health organizations and food manufacturers can take preventative actions by improving food handling procedures or conducting focused inspections because of this predictive capability. According to Smith et al. (2021), the use of predictive analytics in the prevention of foodborne diseases has decreased incident rates by facilitating early interventions and protecting the health of consumers. Monitoring digital platforms, where early indicators of outbreaks frequently appear, requires AI‐driven Natural Language Processing (NLP). Through the examination of news articles, health forums, and social media posts, natural language processing (NLP) models can identify references to food items, symptoms, or places linked to foodborne illness. Frequently, before the filing of formal reports, these algorithms classify and examine the data to find patterns that might indicate an emerging outbreak. According to Zhang and Liu (2020), for instance, real‐time AI monitoring of social media for symptoms of foodborne illness allows for a quicker outbreak response, which helps slow the spread of illnesses. Tracing the sources of contamination within complicated food supply chains is crucial for preventing the spread of foodborne diseases. AI algorithms help with this by evaluating data from various stages of the supply chain to detect the relationships between tainted sources and damaged products. AI‐based traceability solutions can locate contamination sources more accurately than traditional approaches because they can quickly correlate data from supplier records, transit logs, and testing results. Mital et al. (2022) revealed that AI‐enhanced traceability systems improve response times in cases of food contamination, which contributes to the reduction of large‐scale recalls.

Artificial intelligence (AI)‐assisted environmental monitoring helps reduce foodborne infections by keeping manufacturing areas clean. Data from environmental sensors that track parameters such as temperature, humidity, and pathogen levels in facilities that produce food were analyzed using machine learning algorithms. AI systems can assist in stopping the growth and spread of infections by detecting patterns linked to contamination hazards and sending signals for maintenance or sanitary measures. According to research by Kim and Park (2023), contamination rates in food processing plants have significantly decreased owing to AI‐driven environmental monitoring.

**FIGURE 2:**
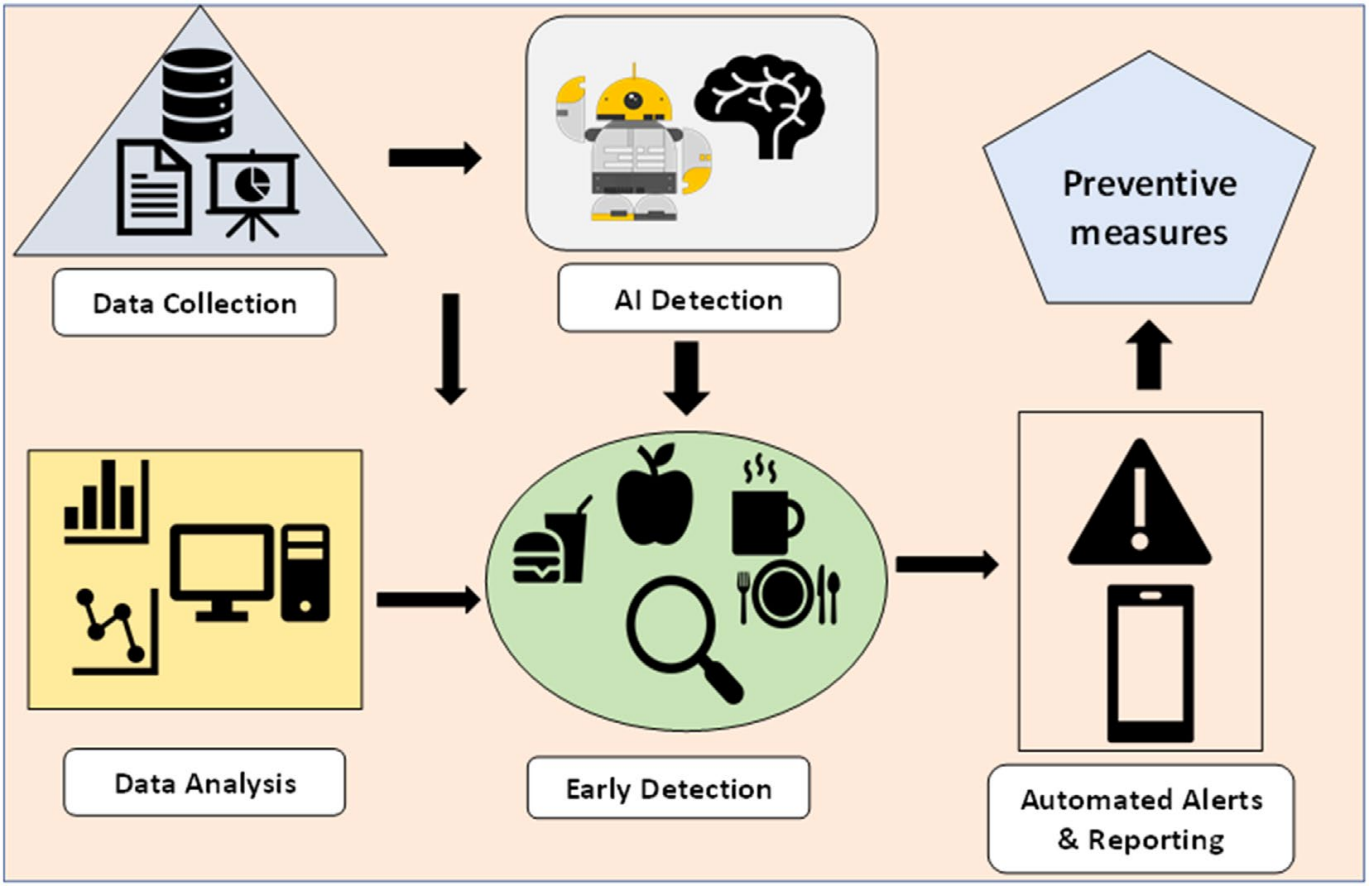
The role of AI in foodborne illness detection and prevention.

AI systems are used to monitor consumer health data to identify trends that might point to an epidemic of foodborne illnesses. AI models can identify anomalous increases in symptoms linked to foodborne infections, such as fever or nausea, by examining patient data, ER visits, and symptom searches on medical websites. Even without test proof, these methods allow public health professionals to quickly detect and address outbreaks. AI‐based health monitoring systems have improved early epidemic identification and lowered the duration and effect of foodborne illness episodes (Brown and Alavi 2021). With tools for predictive analytics, real‐time epidemic identification, contamination tracing, environmental monitoring, and health tracking, artificial intelligence (AI) has revolutionized the field of foodborne illness detection and prevention (Figure 2). Ultimately, these technologies assist in avoiding outbreaks and safeguarding consumers by enabling public health organizations and food producers to take a more proactive approach to addressing food safety issues. The accuracy and effectiveness of foodborne illness prevention measures are continuously being improved by the integration of AI with Internet of Things (IoT) sensors and modern data analytics.

## 11 Quality Control and AI

**FIGURE 3:**
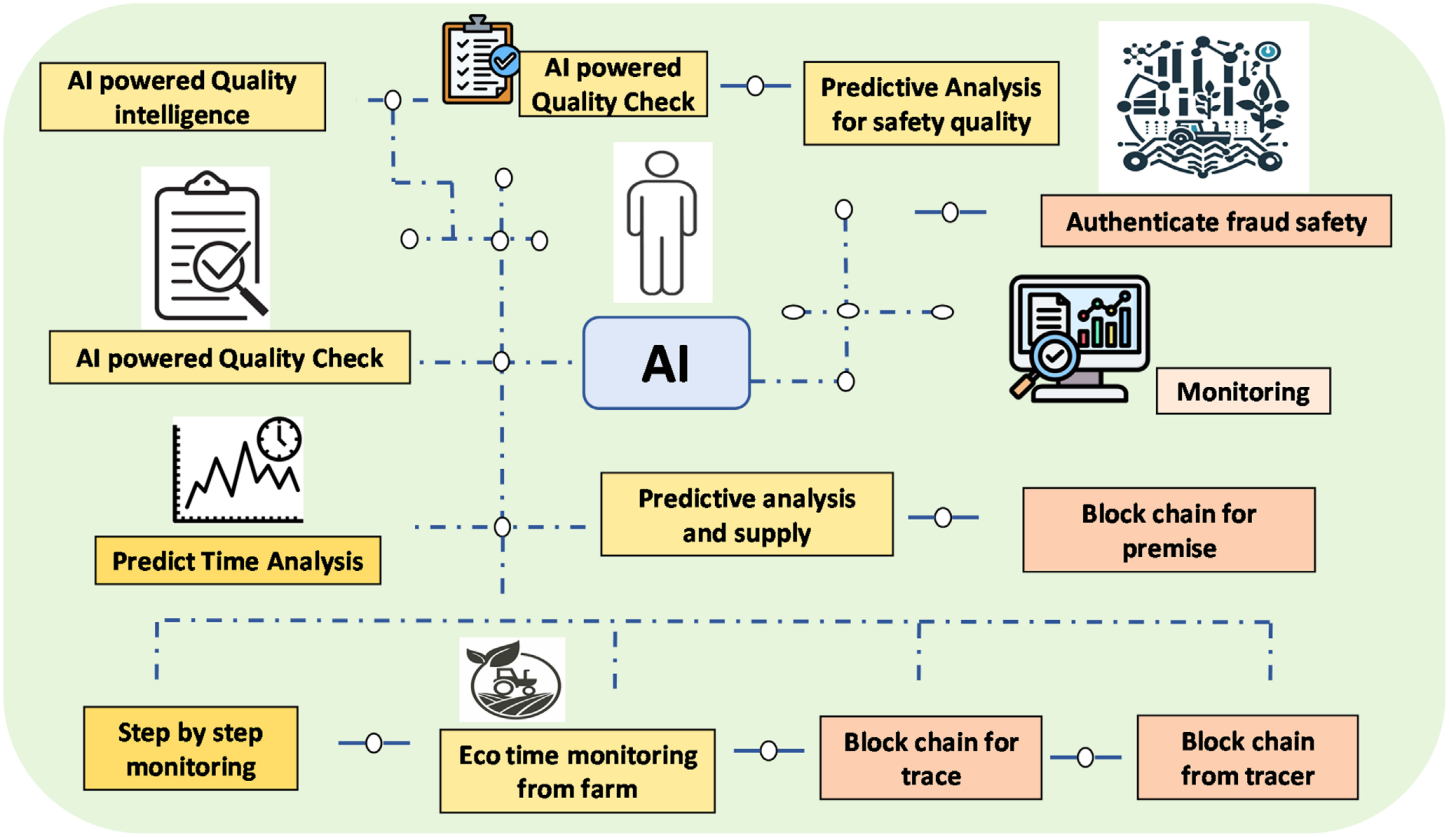
Artificial intelligence (AI) aided technologies in food safety and supply chain.

Quality in the food industry is an exceedingly fundamental aspect that cannot be overlooked and must be embraced with utmost seriousness. They play a crucial role in ensuring consumer safety and satisfaction, both of which are paramount in maintaining public health and trust (Fahle et al. 2020). Traditional quality control measures, which have long been the backbone of these vital industries, are primarily based on the experience and extensive knowledge of experts who have dedicated their careers to maintaining these standards. However, with the advent of technology, specifically artificial intelligence (AI), there has been a significant shift towards automating the quality control process, offering a more modern and innovative approach to ensuring product integrity and excellence (Ribeiro et al. 2021). Automation provided by AI can significantly reduce the risk of human error, which has historically been a major issue in quality assurance. Furthermore, it can improve the overall efficiency of the quality control process in numerous ways, allowing for quicker evaluations and more precise assessments. Various technologies embedded within AI architectures, including advanced machine learning algorithms and sophisticated computer vision techniques, have been utilized for accurate and reliable product quality assessment (Subeesh and Mehta 2021). These technologies work together to analyze products thoroughly, identify defects, and ensure compliance with rigorous industry standards. The primary motivation behind the implementation of these AI applications is to enhance the competitive qualities of their products in a rapidly evolving market landscape (Figure 3). By leveraging AI capabilities, companies can not only streamline operations but also guarantee that their products meet the high standards of quality and reliability demanded by consumers today (Javaid et al. 2021). AI possesses the remarkable capability of generating predictive models and insightful information by meticulously collecting and analyzing vast amounts of data. These data encompass everything from raw materials used in production processes to the final packaging of the product before it reaches consumers (Mökander et al. 2022). Valuable knowledge or models can be meticulously generated from such data, which can subsequently be utilized to support and enhance product quality and safety. Ultimately, the integration of AI into quality control processes represents a significant paradigm shift that promises to revolutionize how food industries operate (Javaid et al. 2021). This transformation is poised to yield benefits that resonate throughout the entire supply chain, improving outcomes not only for manufacturers but also for consumers who rely on these products for their health and well‐being (Zizic et al. 2022).

It has been well‐documented that the direct application of various products utilizing artificial intelligence (AI) has a remarkable potential to significantly enhance both product quality and safety levels across a multitude of industries. Although there may be certain differences in the realms of model development, practical application, and continuous improvements, it is noteworthy that all reported methodologies adhere to a fundamentally similar approach and share an underlying concept (Mavani et al. 2022). AI acts as a vital support mechanism throughout the intricate process of identifying and determining the optimal product quality based on robust, comprehensive, and data‐driven arguments and analyzes. The resulting outputs primarily consist of valuable recommendations and practical guidelines that aid in production processes and enable the accurate prediction of comprehensive product quality profiles (Kumar et al. 2021). Numerous experimental results and well‐documented case studies convincingly demonstrate that artificial intelligence possesses potential that AI can be integrated directly into the food industry, specifically aimed at improving quality and safety standards at a fundamental level. The practical application of these innovative ideas within distinct sectors of the food realm is certain to capture the attention and interest of consumers; the profound potential offered through AI can serve to substantially enhance food safety measures and overall quality levels, addressing critical issues such as contamination and consistency (Kakani et al. 2020; Mavani et al. 2022). Consequently, this particular section of the discussion can serve as an invaluable resource for the enhancement and continual improvement of qualitative data, primarily through the integration of AI into traditional methodologies and practices. In conclusion, the narrative will touch upon future challenges and emerging opportunities that lie ahead in this exciting and rapidly evolving field, inviting further exploration and dialogue on the intersection of AI and food safety initiatives. As we look forward, the ongoing integration of cutting‐edge technologies promises to reshape and redefine industry standards, ushering in a new era of unparalleled efficiency and reliability in food production (Kumar et al. 2021; Ben Ayed and Hanana 2021).

## 12 Regulatory Frameworks for AI in the Food Industry

The incorporation of AI technologies. The primary motivation behind implementing these AI applications is to enhance the competitive qualities of their products in a rapidly evolving market landscape. The vast food value chain presents a remarkable and significant array of benefits that extends from the agricultural sector to various consumer dining experiences. This development not only highlights the immense potential for enhanced productivity levels across the industry but also promotes environmental sustainability and significantly contributes to the reduction of food waste throughout distribution networks (Kumar et al. 2021). Such enhancements can ultimately lead to a much larger volume of food becoming accessible to consumers globally, providing them with a wider variety of choices and potentially improving their quality of life. However, in tandem with these encouraging advantages, there are potential risks that cannot be overlooked, particularly those concerning food safety and quality. Therefore, it is essential to recognize and address these risks appropriately. To establish a robust and comprehensive regulatory framework that governs the effective use of AI systems dedicated to ensuring both the safety and efficacy of foods and animal feeds, it is crucial to thoroughly identify and effectively manage these issues (Sahni et al. 2021). This process necessitates the creation of international and national standards that can effectively address these widespread and critical issues. Overall, while AI technology holds the promise of positively transforming the food value chain, careful consideration and action must be taken to mitigate any associated risks (Ben Ayed and Hanana 2021). Equally important is the recognized and validated implementation of AI technologies and systems within the quality assurance processes of agri‐food products, as this will be instrumental in fostering greater confidence among both consumers and producers regarding their effective use. Recently, policymakers, various stakeholders, and consumers within the agri‐food sector are increasingly seeking comprehensive guidance from national and international food safety authorities, aiming for the most up‐to‐date scientific evidence and regulatory recommendations available (Taneja et al. 2023). To ensure the protection of informed decision‐making processes for consumers on a global scale, there is an urgent need to devise robust food safety regulatory frameworks and efficient risk management systems that not only embrace the numerous opportunities presented by AI, but also diligently confront the multifaceted challenges that arise from its rapid advancements and innovations (Rejeb et al. 2022; Nath et al. 2024).

**FIGURE 4:**
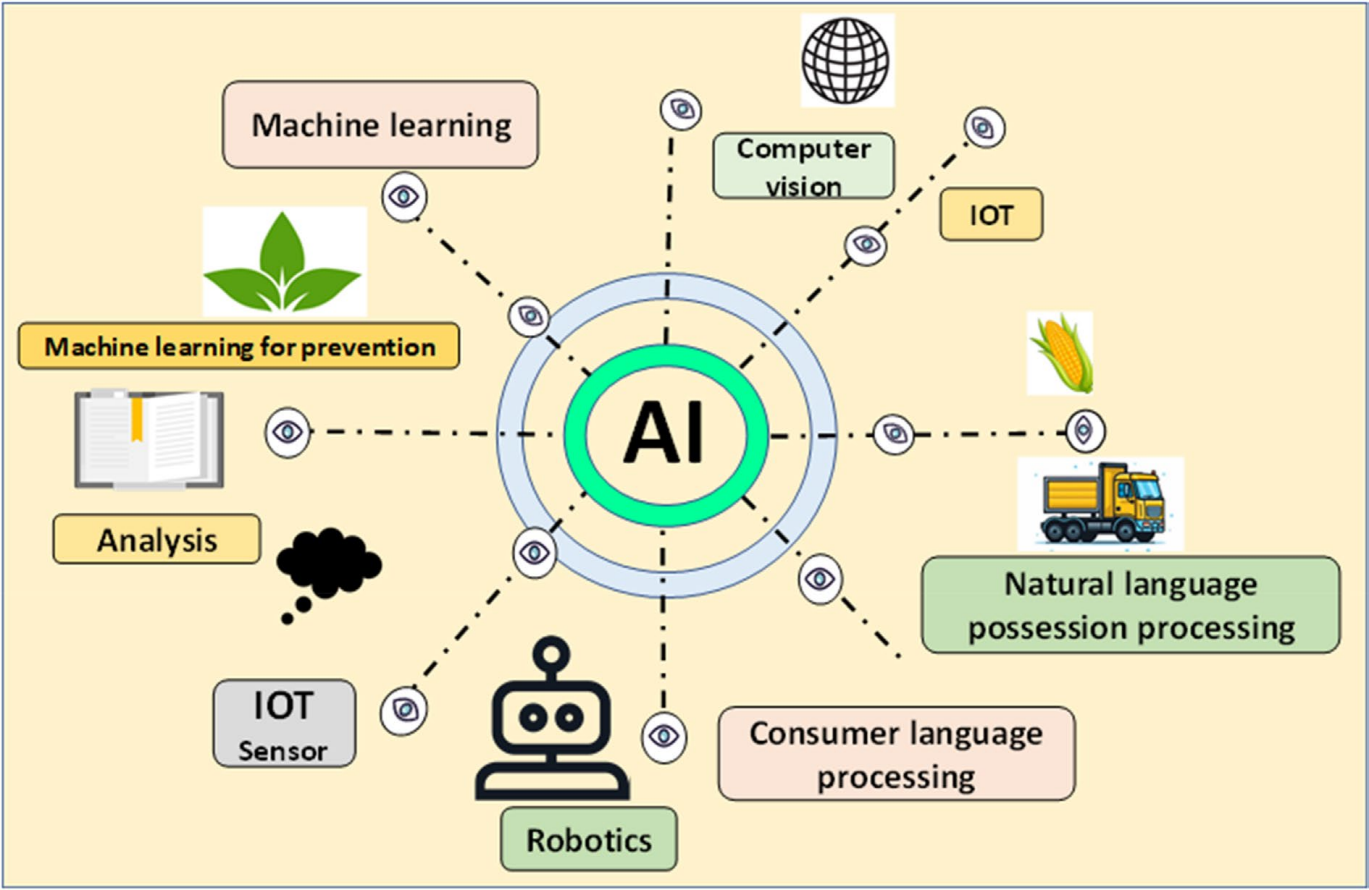
Components of AI as a tool for assessing the quality of various food and agricultural products.

Although there are some existing regulatory approaches within the domain of artificial intelligence, including a somewhat comprehensive set of guidelines and regulations concerning the deployment and management of AI technologies, these frameworks often fail to specifically address or tackle the unique requirements that arise from their application within the critical areas of food safety and quality assurance (Falco et al. 2021). Active dialogue and consistent engagement with various stakeholders throughout the entire risk management process are essential for fostering societal acceptance as well as ensuring market readiness for new technologies (Figure 4). A thorough gap analysis examining the current regulatory landscape concerning AI in the food and feed sectors is vital, particularly given the rapid pace at which AI technologies are evolving and advancing (Manning et al. 2022). Moreover, strict adherence to established food safety regulatory frameworks is critical for businesses operating in the agri‐food industry, and any significant failures in compliance could result in severe enforcement actions, potentially involving civil or even criminal penalties that could adversely affect their operations and overall business standing (Díaz‐Rodríguez et al. 2023).

## 13 Ethical Considerations in AI Applications for Food Safety

It is primarily focused on the synthesis of datasets but is specifically centered on the real‐world applications of artificial intelligence in the realms of food safety and quality assurance. However, for reasons that should be clear to the reader at this point, it is essential to critically inquire into some critical factors, including the ethical obligations that producers of AI‐based systems must uphold and the mechanisms of these AI‐based systems to operate according to established ethical principles. This exploration is especially pertinent in the context of the ultimate goal of leveraging AI to enhance food safety, which is fundamentally aimed at preventing unnecessary harm arising from foodborne illnesses (Nayak and Dutta 2023). Nonetheless, if not sufficiently careful and conscientious in both the development and implementation phases of AI technology, there might be ethically unacceptable outcomes that could manifest in a variety of ways, including an increase in the incidence of foodborne illnesses (Manning et al. 2022).

The most obvious concern about utilizing AI technologies to detect and prevent various food contaminants is that a probability can be articulated straightforwardly. The probability of contamination in a cargo shipment or food sample presented to a machine‐learning system is exceedingly low to the point where it can be regarded as virtually zero, which means that the ramifications of multiple false rejections must be assessed (Kudashkina et al. 2022). Such instances could potentially lead an AI‐driven food safety system to endure a catastrophic moment, resulting in effects that could significantly damage the trust that the industry places in AI technology. These biases regarding possible food contamination are not necessarily inherent to the data itself, nor are they fundamental flaws in the algorithm employed. Instead, these issues predominantly stem from the training data; unfortunately, when it comes to the application of AI in real‐world scenarios, this critical aspect often goes unchecked and remains unaddressed (Geng et al. 2022). Furthermore, the existence of methodologies that lack sufficient transparency or accountability means that most AI predictions, including the urgent and pressing goals of scanning for emerging types of pathogens, are far too hazardous to be implemented outside of a controlled environment. This represents a notably unintelligent strategy for utilization in the critical areas of food safety (Liu et al. 2022). A number of ethical concerns are sharply illuminated by media portrayals of AI being used inappropriately, in an ambiguous manner, or both, which can exacerbate the anxieties surrounding its use. The implications of this can have far‐reaching effects, particularly when consumers and industry stakeholders alike begin to question the integrity and reliability of AI systems to ensure food safety standards (Addanki et al. 2022).

## 14 Case Studies in AI Implementation for Food Safety and Quality Assurance

Artificial Intelligence (AI) has shown encouraging results in food safety and quality assurance (QA), improving food firms' capacity to identify contamination, track compliance, and guarantee high quality across the supply chain. Case studies from a range of food business sectors have shown how AI has been effectively used to solve certain problems, indicating its potential to revolutionize conventional QA procedures. This section examines several noteworthy case studies that demonstrate the use of AI in regulatory compliance, traceability, contamination detection, and quality control. One notable example is the application of AI for real‐time quality monitoring of dairy production. A major dairy producer used AI‐powered sensor systems in conjunction with machine‐learning algorithms to check milk quality throughout the processing steps. The AI system evaluated temperature, pH levels, and bacterial counts, notifying operators in real‐time of any departure from quality standards. This approach reduced spoilage by 25%, while increasing the overall quality consistency by 30% (Martin et al. 2022). This case study highlights how AI can maintain high‐quality standards while reducing waste in perishable food production, where timing is critical.

In a different case study, an AI‐based picture recognition system was implemented by a meat processing company to identify indications of contamination in manufacturing lines. The system identified possible problems, including discolourations, foreign items, or inconsistencies in texture, by analyzing high‐resolution photographs of beef products using computer vision technology. Robotic arms and AI‐driven image processing were used to immediately remove any suspected products from the production line. Chen and Wu (2021) claimed that this system's contamination detection rate of over 98% greatly reduces the possibility that tainted meat would reach consumers and eliminates the need for labor‐intensive manual inspections.

A beverage manufacturing company has difficulty adhering to severe food safety rules across various facilities. The organization uses an AI‐based document processing system to automate the evaluation of compliance records, internal audits, and regulatory standards. NLP algorithms retrieve essential data from inspection reports and compare them with regulatory criteria, highlighting non‐compliant entries for review. According to Roberts et al. (2023), this method lowered audit preparation time by 40% while minimizing compliance errors. This instance demonstrates how AI may improve regulatory compliance processes, especially in businesses with complicated and frequent audits. The intricate supply chains of the seafood industry make it especially susceptible to problems such as fraud, mislabeling, and contamination. A seafood supplier combined AI and blockchain technology to improve the traceability from fishing boats to retail locations. The AI component evaluates supplier data, shipment records, and testing reports to verify product origins and quality standards, whereas the blockchain provides a secure, unchangeable record of each transaction. This solution increased traceability accuracy by 45% while reducing fraud by 30% (Nguyen and Lin 2021). This instance demonstrates how AI and blockchain can provide transparency in supply chains, which is crucial for high‐risk food categories. Fresh produce is vulnerable to contamination by bacteria, such as *E. coli* , which can cause extensive outbreaks if not caught early. A multinational agricultural corporation has built an artificial intelligence system that analyzes social media, news sites, and health forums for discussions on foodborne illness symptoms. AI uses natural language processing (NLP) to identify references of specific produce items, symptoms, and locations, allowing for the early detection of possible epidemics. When the system detected an increase in gastrointestinal complaints within one of the company's distribution areas, preventive measures were implemented, thereby controlling a potential outbreak. Carter et al. (2020) presented a case study that showed how AI in social media monitoring might improve response times and reduce public health hazards in the fresh produce market. The efficiency of AI in real‐time quality control, contamination detection, automated compliance, traceability, and outbreak identification is demonstrated by these case studies, which highlight the many uses of AI in food safety and quality assurance. Every example shows how AI can increase precision, reduce human error, and simplify intricate procedures in the distribution and manufacture of food. The use of AI technologies in food safety and quality assurance is anticipated to grow as they develop further, offering increasingly advanced solutions for risk management and regulatory compliance.

## 15 Concluding Remark

AI‐based technologies play a vital role in the food processing sector, from farms to forks. The application of AI in ensuring food safety with higher quality is an important aspect of the Industrial 4.0 revolution from a consumer perspective. AI‐based technologies such as machine learning, computer vision, and blockchain allow one to keep a close watch on the product and detect the presence of contaminants in real time, analyze the data, and take timely action that will help to eliminate foodborne illness. There has been continuous growth and progress in AI‐based technologies, as evident from the research discussed in this review. This will help in implementing AI‐based technologies at an industrial scale to ensure the delivery of safe food to consumers worldwide. However, future research still needs to focus on developing AI algorithms and sensors that are consumer‐friendly and can be used by consumers to assess the freshness of the product before it is consumed. The sensors should be able to convey the freshness of the product to consumers. With the help of AI‐based technologies, the quality and safety of food products can be improved, and the food industry can obtain real‐time data.

## Author Contributions

**Harsh B. Jadhav:** conceptualization, investigation, writing – original draft, writing – review and editing, software, data curation. **Moumita Das:** writing – original draft, writing – review and editing, data curation, validation. **Arpita Das:** validation, writing – original draft, writing – review and editing, data curation. **Aayeena Altaf:** data curation, writing – review and editing, writing – original draft, validation. **Kamal Alaskar:** writing – review and editing, validation, writing – original draft, data curation. **Robert Mugabi:** writing – review and editing, data curation, writing – original draft.

## Funding

The authors have nothing to report.

## Disclosure

The authors have nothing to report.

## Ethics Statement

The authors have nothing to report.

## Consent

All the authors contributed substantially to the development of the manuscript and approved the final submission.

## Conflicts of Interest

The authors declare no conflicts of interest.

## Data Availability

The data that support the findings of this study are available from the corresponding author upon reasonable request.
